# Assessment of Knowledge, Attitudes, Prevention Practices, and Self‐Reported Malaria Prevalence Among Summer Degree Students at Debre Markos University, Ethiopia, 2025

**DOI:** 10.1155/japr/5834041

**Published:** 2026-06-16

**Authors:** Asmamaw Habtamu, Lamenew Fenta

**Affiliations:** ^1^ Department of Biology, Debre Markos University, Debre Markos, Ethiopia, dmu.edu.et

**Keywords:** Debre Markos University, Ethiopia, insecticide-treated nets, knowledge attitudes and practices, malaria prevalence

## Abstract

**Background:**

Malaria remains a major public health problem in Ethiopia, affecting all population groups including educated professionals such as university students. Understanding knowledge, attitudes, and prevention practices among these study participants is crucial for designing effective interventions. This study is aimed at assessing self‐report prevalence, knowledge, attitude, prevention practices, and related factors of malaria among summer students pursuing bachelor′s degrees at Debre Markos University from July to September 2025.

**Methods:**

An institution‐based cross‐sectional study was conducted among 366 study participants using a structured questionnaire to collect data on sociodemographic characteristics and malaria‐related knowledge, attitudes, prevention practices, and its self‐report prevalence. Data were analyzed using bivariate and multivariate logistic regression to identify factors associated with malaria prevalence.

**Results:**

Among 366 study participants, 63.7% were male, and 50.83% were aged 30–39 years. The majority (70.5%) resided in rural areas of the East Gojjam, West Gojjam, and Awi zones, as well as areas surrounding Bahir Dar City in the Amhara Region. Overall, 16.7% of participants tested positive for malaria between April and June 2025. Regarding knowledge, 75.4% demonstrated satisfactory awareness of malaria, and 82.8% correctly identified its causative agent. However, knowledge about insecticide‐treated net (ITN) utilization and appropriate treatment‐seeking behavior was limited. In terms of attitudes, 74.86% recognized malaria as a serious health problem, and 71.04% agreed on the importance of ITN use. Despite this, preventive practices were inadequate: only 71.58% reported consistent ITN use, 39.07% sought treatment at health facilities, and 15.57% completed the full course of antimalarial medication. Rural residence, inconsistent ITN use, and failure to eliminate mosquito breeding sites were significantly associated with malaria prevalence (*p* < 0.05).

**Conclusion:**

Malaria remains a significant public health concern even among educated populations in the study area. The findings highlight gaps in prevention practices and treatment compliance despite good knowledge and attitudes. Strengthening behavioral interventions, promoting consistent ITN use, and enhancing community‐based awareness programs are essential to reduce malaria transmission among university students and rural communities.

## 1. Introduction

Malaria is a life‐threatening disease that is caused by *Plasmodium* parasites and transmitted to humans through the bites of infected female Anopheles mosquitoes [1]. It remains a major public health challenge, particularly in tropical and subtropical regions. According to the World Health Organization, an estimated 263 million cases and 597,000 deaths were reported globally in 2023, with Africa accounting for about 94% of these cases [2]. Ethiopia is among the high‐burden countries, where over 60% of the population is at risk of infection, with seasonal peaks following the rainy season [3]. In recent decades, malaria has caused several epidemics in different parts of the country, particularly in lowland and semi‐highland areas [4].

The National Malaria Elimination Program of Ethiopia (NMEP) has strengthened malaria control through several key interventions. These include widespread distribution of insecticide‐treated nets (ITNs), targeted indoor residual spraying (IRS), universal access to early diagnosis and prompt treatment, and community health education. These strategies are outlined in the National Malaria Elimination Strategic Plan 2021–2025. These interventions have significantly reduced malaria prevalence [3, 5], and malaria persists as a major health problem due to sociobehavioral factors such as poor adherence to preventive interventions.

Attitudes have a significant influence on the development of preventive health behaviors. An attitude refers to a person′s feelings, beliefs, or predispositions toward a behavior or situation [6]. Consistent use of ITNs, early treatment‐seeking, and participation in community awareness activities are encouraged by positive attitudes in malaria prevention. In contrast, negative or indifferent attitudes contribute to poor uptake of preventive measures and sustained transmission. [7]. Health behavior theories, such as the health belief model (HBM), emphasize that attitudes are influenced by perceptions of susceptibility, severity, benefits, and barriers, which all have a direct effect on preventive practices [8]. Understanding these attitudes is therefore essential for creating effective health interventions [9]. Evidence showed that designing tailored health education interventions that strengthen both individual protection and community‐wide malaria prevention [7].

Knowledge plays a critical role in shaping preventive behaviors against malaria. This encompasses awareness of the etiological agent, recognition of common clinical manifestations such as fever, chills, and headache, and understanding that malaria is primarily transmitted through the bites of infected mosquitoes. Adequate knowledge of malaria transmission, symptoms, and prevention methods has been consistently associated with improved adoption of preventive measures, including ITN utilization and appropriate health‐seeking behavior. Conversely, misconceptions and limited awareness regarding malaria transmission and prevention reduce participation in control programs and increase vulnerability to infection [9].

Summer degree students constitute an important group to assess malaria situations in this study area. At Debre Markos University, they represent a mobile and diverse population, coming from both malaria‐endemic and nonendemic areas. Their varied experiences shape their perceptions and practices. As future community leaders, these students can act as change agents by promoting awareness and encouraging preventive practices in their home communities and among students in their school. However, little is known about their level of knowledge, attitudes, and practices (KAP) regarding malaria prevention, which raises concern in terms of sustaining effective control efforts.

Although Ethiopia has scaled up preventive measures such as ITN distribution and awareness campaigns, their success largely depends on the KAP of the population. Evidence indicates that, despite relatively high awareness of malaria symptoms, consistent adoption of preventive measures such as ITN use, environmental sanitation, and timely treatment‐seeking remains inadequate such gaps compromise malaria control strategies and contribute to continued transmission. Therefore, assessing the knowledge, attitudes, practices, and self‐malaria prevalence report of summer students toward malaria at Debre Markos University is crucial.

## 2. Methods

### 2.1. Description of the Study Area

**Figure 1:**
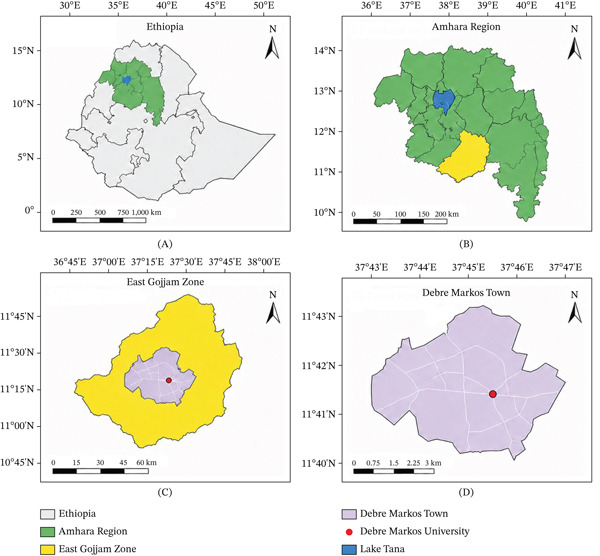
Map of the study area.

**Figure 2:**
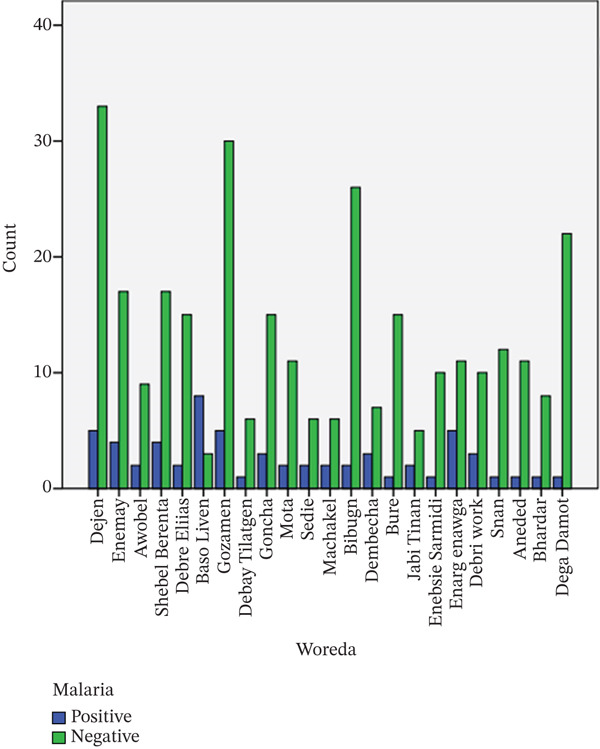
Bar graph showing malaria cases among study participants from different woredas, April–June 2025.

The study was conducted in Debre Markos University, East Gojjam, Amhara National Regional state, Ethiopia (Figure 1 and 2). Debre Markos, the capital city of East Gojjam, is located 299 km northwest of Addis Ababa, with an altitude ranging from 2420 to 2509 m (8000 to 8230 feet) above sea level, with an average temperature of approximately 17°C–21°C and an average annual rainfall of approximately between 1200 and 1380 mm.

### 2.2. Study Design

An institution‐based cross‐sectional study was conducted from July to September 2025 to assess the levels of attitudes and practices toward malaria among summer students attending Debre Markos University. Participants were also requested to self‐report malaria occurrences during the preceding 3 months (April to June 2025) to capture recent malaria experiences before the peak transmission period.

### 2.3. Study Population

The study population comprised all summer students enrolled during the study period. Participants were also asked to self‐report any malaria episodes experienced during the preceding 3 months (April to June 2025), which was intended to capture recent malaria history prior to the peak transmission season and minimize recall bias.

### 2.4. Sample Size Determination and Sampling Technique

The sample size was determined using a single population proportion formula by taking the proportion with a confidence interval of 95%, with 5% margin of error, and 5% nonresponse by using the formula [10]:

n=Z2∗p∗1−p/d²,

where where *n* is the required sample size; *z* is the standard normal distribution value at a 95% confidence level (*z* = 1.96); *p* is the estimated prevalence of malaria; because no previous similar study was available in the study area, 50% (*p* = 0.5) was used to obtain the maximum sample size; and *d* = margin of error, set at 5% (*d* = 0.05). So, 384 was the required sample size for a large (infinite) population, but for a finite population, the correction formula was used. *n* = *n* *o*/(1 + (*n* − 1/*N*)). The sample is 349; adding 5% nonrespondent rate, the final sample was 366.

Multistage stratified sampling techniques were employed to select the study population. First, 30% of the colleges and academies registering summer degree students were randomly selected using a rule of thumb. Accordingly, the College of Natural Sciences, the College of Social Sciences, and the Sport Science Academy were included. In the next stage, samples were proportionally allocated among 366 summer degree students from 10 departments: Amharic (73), English (48), Civics (25), Geography (35), History (24), Biology (53), Chemistry (22), Physics (13), Mathematics (22), and Sport Science (51).

### 2.5. Inclusion and Exclusion Criteria

The study population included all summer students enrolled in the 2025 academic year. Students who did not agree to the consent or were absent during data collection were excluded from the study.

### 2.6. Data Collection Methods

Data were collected using a structured questionnaire. The questionnaire covered sociodemographic characteristics, prior malaria history, ITN and IRS use, and care‐seeking practices. It was designed to assess participants′ KAP regarding the causes, modes of transmission, and preventive methods of malaria. The questionnaire was initially prepared in English and then translated into the local language (Amharic) by an expert fluent in both languages to ensure consistency.

The knowledge assessment scores for the participants′ knowledge regarding malaria were determined as follows. A correct answer to a knowledge question was assigned 1, whereas an incorrect or do not know answer was scored 2. The original Bloom′s cutoff points were adapted and modified to a small extent for this study. Accordingly, respondents obtaining 80.0%–100.0% score were classified as having good knowledge, those having 60.0%–79.0% score were categorized as having satisfactory knowledge, and those having ≤ 59.0% score were classified as having poor knowledge [11].

Attitude was quantified using Likert′s scaling technique. Attitude questions that contained positive and negative statements and responses were rated on a 5‐point Likert scale: strongly agree with score of 5, agree with score of 4, undecided with score of 3, disagree with score of 2, and strongly disagree with score of 1. Responses of each subject were summed to obtain a total attitude score. The mean attitude score for all the participants was calculated. The participants who obtained a result of equal to or above the mean were marked as having a positive attitude, and those with a result below the mean were marked as having a negative attitude toward malaria.

The practices of the participants were also scored on a Likert‐type scale. The responses were scored as never (Score 0), sometimes (Score 1), and always (Score 2). The response of every participant was summed, and a total practice score was obtained. The average practice score will be computed for all the participants in the study. An individual was classified as having good practice when his or her overall practice score is on or above the mean, and members whose scores are below the mean were classified as having poor practice.

### 2.7. Data Analysis

The collected data were checked for completeness and consistency before analysis. Data were coded and entered into Microsoft Excel and analyzed using SPSS version 24.0. Questions related to malaria signs and symptoms, mode of transmission, and preventive measures were combined to determine the respondents′ knowledge and attitudes regarding malaria. Descriptive statistics, including frequencies, percentages, means, and standard deviations, were computed to summarize participants′ knowledge levels.

Knowledge scores were categorized as good, satisfactory, or poor based on Bloom′s cutoff points. Likert‐scale responses were summed to produce an overall attitude score for each participant. The mean attitude score was calculated, and participants were categorized as having a positive or negative attitude depending on whether their score was equal to or above, or below, the mean. Descriptive statistics were presented, and associations with independent variables were assessed using bivariate logistic regression. Variables with *p* values of < 0.25 in bivariate analysis were included in a multivariable logistic regression model to control for potential confounders and to determine independent predictors of knowledge, attitude, and practice levels.

### 2.8. Ethical Consideration

Ethical clearance was obtained from the Department of Biology, Debre Markos University, and the consent agreement was signed by the researcher and the study participants prior to data collection.

### 2.9. Data Quality Assurance

The questionnaires were pretested on 5% of the sample before the start of actual data collection. Each questionnaire was reviewed to verify that all required fields had been completed correctly and labeled with the participants′ identification numbers to prevent result mix‐ups. Slides were cross‐checked.

## 3. Results

### 3.1. Sociodemographic Characteristics of the Study Participants

Among the total of 366 participants, 63.7% of them were males, and the rest (36.3%) were females, with the majority (50.83%) age ranging from 30 to 39 years old. Additionally, most of the study participants (70.5%) came from rural areas of the east, west, and Awi zone and around the cities of the Amhara Region; 16.7% of the study participants were positive with blood examination from April to June 2025, as presented in Table 1.

**Table 1:** Sociodemographic characteristics of the study participants from July to September 2025.

Variables	Category	Frequency	Percent
Age	20–29	150	40.98
	30–39	186	50.83
	40–49	29	7.92
	≥ 50	1	0.27
Sex	Male	233	63.7
	Female	133	36.3
Department of the study participants	Biology	53	14.5
	Chemistry	22	6
	Physics	13	3.6
	Mathematics	22	6
	Amharic	73	19.9
	English	48	13.1
	History	24	6.6
	Geography	35	9.6
	Civics	25	6.8
	Health and Physical Education	51	13.9
Residence	Urban	108	29.5
	Rural	258	70.5
Marital status	Single	147	40.2
	Married	215	58.7
	Divorced	4	1.1
Malaria status from April to June 2025	Positive	61	16.7
	Negative	305	83.3

As indicated in Figure 1, the largest number of participants (39) were from Dejen district, and the least (7) from Debay Tilatgen and Jabi Tihnan districts. Additionally, a higher proportion of malaria infection (72.7%) and the lowest malaria infection (4.3) were recorded from Baso Liven and Dega Damot districts, respectively.

### 3.2. Knowledge of Study Participants on Malaria

Regarding knowledge of malaria, 75.4% of the study participants demonstrated a satisfactory level of knowledge according to Bloom′s cutoff. Furthermore, 82.8% of participants showed a good understanding of the causes of malaria. In contrast, participants′ knowledge regarding ITN utilization and appropriate care‐seeking during infection was poor, as shown in Table 2.

**Table 2:** Knowledge of study participants on malaria, 2025.

Variables	Category	Frequency	Percent
Knowledge of malaria?	Yes	276	75.4
	No	90	24.6

Cause of malaria	Protozoa	303	82.8
	Bacteria	53	14.5
	Bad air	7	1.9
	Other	3	0.8
Transmission of malaria	Mosquito bite	326	89.1
	From person to person	12	3.3
	Through contaminated drink/food	9	2.5
	Do not know	19	5.2

Can malaria be prevented?	Yes	302	82.5
	No	41	11.2
	I do not know	23	6.3
Malaria methods	ITNS use	159	43.44
	IRS	21	5.74
	Elimination of breed site	71	19.4
	Use of traditional remedy	11	3.01
	All	93	25.4
	I do not know	11	3.01

Where to treat malaria	Health facility	201	54.9
	Pharmacy	50	13.7
	Traditional healer	17	4.6
	Home remedy	45	12.3
	Do not know	46	12.6
	All	7	1.9

### 3.3. Attitudes of the Study Participants Toward Malaria Prevention

Of the participants, 74.86% had a positive attitude, and 25.14 had a negative attitude toward the seriousness of malaria in the study. On the contrary, only 53.01% of the study participants had a positive attitude toward the distribution of malaria in the study participants, as indicated in Table 3

**Table 3:** Attitudes of the study participants toward malaria prevention, 2025.

Variables	Category	Frequency	Percent
Seriousness of malaria	Positive attitude	274	74.86
	Negative attitude	92	25.14
Distribution of malaria	Positive attitude	194	53.01
	Negative attitude	172	49.99
Use of ITNs to prevent malaria	Positive attitude	260	71.04
	Negative attitude	106	28.96
IRS use to prevent malaria	Positive attitude	219	59.84
	Negative attitude	147	40.16
Use of traditional medicine	Positive attitude	205	56.01
	Negative attitude	161	43.99
Early diagnosis can prevent malaria.	Positive attitude	228	62.3
	Negative attitude	138	37.70
Everyone is responsible for preventing malaria.	Positive attitude	212	57.92
	Negative attitude	154	42.08

### 3.4. Prevention Practices of the Study Participants

Regarding malaria prevention practices, 7.58% of the participants performed a good utilization of INTs to prevent malaria in the study participants, whereas only 15.57% and 39.07% of the study samples had performed good practices on completion of the full dose of antimalarial treatment and visiting the proper places to treat malaria, respectively (Table 4).

**Table 4:** Prevention practice of malaria among study participants in 2025.

Variables	Category	Frequency	Percent
Use of INTs last night	Good practice	262	71.58
	Poor practice	104	28.42
Elimination of the breeding site	Good practice	289	78.98
	Poor practice	77	21.02
Proper place for seeking to treat malaria	Good practice	143	39.07
	Poor practice	223	60.93
Completion of dose during chemical therapy	Good practice	57	15.57
	Poor practice	309	84.43
How often use IRS	Good practice	110	30.05
	Poor practice	256	69.95

### 3.5. Health Facility–Related Factors of the Study Participants

Most study participants (80.3%) reported having a nearby health facility, whereas 19.7% did not. Available facilities included health centers (35.5%), hospitals (29.0%), drug shops (20.2%), and private clinics (15.3%). When seeking care, 32.2% visited government facilities; 23.2%, pharmacies; 24.9%, traditional healers; and 19.7%, private clinics. Malaria drugs were reported to be available by 67.2% of participants, unavailable by 21.3%, and unknown by 11.5%. Regarding affordability, 53.8% could afford care, 38.0% could not, and 8.2% were unsure (Table 5).

**Table 5:** Health facility–related factors of the study participants, 2025.

Variables	Category	Frequency	Percent
Availability of a nearby health facility	Yes	294	80.3
	No	72	19.7

Distance of the health facility from home on foot walk	Less than 30 min	78	21.3
	b/n 30–60 min	103	28.1
	b/n 61–120 min	139	38.0
	More than 120 min	46	12.6

Presence of health facilities to visit	Hospital	106	29.0
	Health center	130	35.5
	Private clinic	56	15.3
	Drug shop	74	20.2

The health institute to seek care	Government health facility	118	32.2
	Private clinics	72	19.7
	Pharmacy	85	23.2
	Traditional healer	91	24.9

Type diagnosis to screen malaria	RDT	105	28.7
	Microscopy	124	33.9
	Clinical signs	94	26.5
	Not known	40	10.9

Availability of malaria drugs in the health facilities	Yes	246	67.2
	No	78	21.3
	Not known	42	11.5

Affordability	Yes	197	53.8
	No	139	38.0
	Not known	30	8.20

ITNs distributed to the community	Yes	174	47.57
	No	192	52.43

IRS employed at community houses	Yes	144	39.34
	No	222	60.67

### 3.6. Bivariate and Multivariate Logistic Regression of Factors on Malaria

Bivariate and multivariate logistic regression indicated that the study participants living in the rural area showed significantly higher malaria cases than those living in the urban area. Similarly, INT utilization and mosquito breeding site elimination showed a significant association with the prevalence of malaria (Table 6).

**Table 6:** Bivariate and multivariate logistic regression of potential factors of malaria among the study participants, 2025.

Variable	Category	Malaria prevalence		Bivariate logistic regression			Multivariate logistic		
		Positive (%)	Negative (%)	COR	CI	*p* value	AOR	CI	*p* value
Residence	Urban	11.1	89.9	1.9	[0.9, 3.9]	0.06	2.3	[1.02, 5.2]	0.039∗
	Rural	22.8	77.2						

Woreda	Baso Liven	72.7	27.3	58.7	[5.3, 649]	0.001	71.4	[4.5, 114.5]	0.003∗
	Sedie	25	75	7.33	[0.5, 6.95]	0.128	17.6	[1.04, 297]	0.046∗
	Dembecha	30	70	9.4	[0.84, 105.8]	0.069	21.9	[1.4, 348]	0.026∗
	Jabi Tinan	28.5	81.5	8.8	[067, 117.2]	0.1	31.3	[1.7, 568]	0.02∗
	Enarg Enawga	31.25	78.75	10	[1.04, 96.4]	0.046	30.34	[2.5, 388]	0.009∗
	Dega Damot	4.3	95.7						

Use of INT	Always	11.5	89.5	4	[1.9, 8.4]	0.001	12.7	[4.1, 39]	0.001∗
	Sometimes	17.1	82.9	4.1	[2.1, 7.8]	0.001	5.7	[2.5, 13]	0.001∗
	Never	34.1	63.9						

Elimination of the breeding site	Yes	15.5	84.5	2.6	[1.5, 4.7]	0.002	3.8	[1.6, 8.9]	0.002∗
	No	28.5	71.5						

Near health facility	Yes	20.5	79.5	1.1	[0.6, 2.3]	0.72			
	No	15.3	84.7						

Availability of malaria drug	Yes	19.1	80.9	0.44	[0.15, 1.3]	0.14	0.67	[0.16, 2.78]	0.57
	No	12.82	87.18	0.71	[0.21, 2.44]	0.59			0.93
	Not known	9.5	91.5						

Affordability of drug	Yes	19.7	80.3	1.4	[0.54, 3.9]	0.46			
	No	18.7	81.3	1.1	[0.4, 2.9]	0.87			
	Not known	20.5	79.5						

*Note:* Asterisk (^∗^) means statistically significant association at *p* ≤ 0.05.

Abbreviations: AOR, adjusted odds ratio; CI, confidence interval; COR, crude odds ratio; INT, insecticide‐treated net.

## 4. Discussion

The sociodemographic characteristics of the 366 study participants indicated that males constituted a larger proportion (63.7%) compared to females (36.3%). This gender distribution is consistent with previous Ethiopian studies reporting higher male participation in community‐based health research, which may relate to increased mobility and outdoor activities like education among men, exposing them more frequently to malaria vectors [12]. Age analysis revealed that most participants were between 30 and 39 years (50.83%), followed by those aged 20–29 years (40.98%), suggesting a predominance of economically and educationally active individuals. These age groups are often engaged in outdoor occupations such as education, farming, and construction, which heighten malaria exposure risk [13].

Regarding residence, 70.5% of participants were from rural areas, compared to 29.5% from urban settings. This aligns with national malaria patterns, where rural regions typically experience higher transmission due to proximity to mosquito breeding sites and limited access to preventive measures such as ITNs and IRS (FMOH, 2023). Regarding academic diversity, the higher representations were taken from Amharic (19.9%) and Biology (14.5%) departments based on their population size reflecting the interdisciplinary nature of health and environmental awareness in the region. This diversity likely enhances the reliability of findings by integrating perspectives from multiple fields.

Marital status data indicated that 58.7% of participants were married, 40.2% were single, and 1.1% were divorced. Marital status may influence malaria exposure, as married individuals often reside permanently in rural areas, increasing their contact with vector habitats, whereas single individuals may be more mobile or urban‐based [14]. Social behaviors related to marital status, such as sleeping arrangements and household prevention practices, could further affect vulnerability to infection.

Laboratory results showed that 16.7% of participants tested positive for malaria between April and June 2025, reflecting ongoing transmission during the rainy season when mosquito breeding is favorable [2]. The detection of malaria among educated individuals underscores that awareness alone does not ensure prevention, highlighting the continued need for vector control, surveillance, and targeted health education [15].

Malaria prevalence varied geographically across woredas (districts). Baso Liven district reported the highest infection rate (72.7%), likely due to favorable ecological conditions, including stagnant water and irrigation practices [13]. In contrast, Dega Damot district had the lowest prevalence (4.3%), which may be associated with higher altitude and cooler temperatures that limit mosquito populations [3]. These differences emphasize the localized nature of malaria transmission and the necessity for area‐specific interventions.

Knowledge assessment revealed that 75.4% of participants had satisfactory knowledge about malaria based on Bloom′s cutoff, with 82.8% identifying protozoa as the causative agent and 89.1% recognizing mosquito bites as the primary transmission route. These findings are consistent with prior studies in Northwest Ethiopia, where awareness campaigns and school‐based education programs improved understanding of malaria [15]. Despite this, gaps persisted: only 43.4% reported using ITNs, and 19.4% practiced mosquito breeding site elimination. These gaps between knowledge and practice are echoed in other Ethiopian studies and may result from net misuse, inadequate distribution, or misconceptions regarding preventive tools [12]. Health‐seeking behaviors also varied, with 54.9% seeking treatment at health facilities and others relying on pharmacies (13.7%), home remedies (12.3%), or traditional healers (4.6%), indicating that cultural practices and accessibility issues continue to influence care‐seeking [3]. This emphasizes the need for education programs that promote timely utilization of health services alongside community engagement to improve preventive behaviors [2].

Regarding attitude, 74.86% of participants had a good attitude that recognized malaria as a serious health threat, which aligns with studies from Ethiopia and Nigeria demonstrating high awareness of malaria severity [16, 17]. However, only 53.01% of the participants had a good attitude to appreciate the widespread distribution of malaria, suggesting a gap in understanding local transmission patterns, which may limit preventive efforts [18]. Attitudes toward preventive measures varied: 71.04% had a positive view of ITN use, 59.84% supported IRS, and lower percentages favored traditional medicine (56.01%) or community responsibility (57.92%). However, promoting positive attitudes across all preventive measures remains crucial for effective malaria control [19].

Regarding malaria prevention practices, the present study revealed that 71.58% of participants reported good utilization of ITNs, which aligns with findings from northwest Ethiopia, where ITN ownership and use were moderate but inconsistent [19, 20]. Additionally, 78.98% engaged in mosquito breeding site elimination, consistent with findings showing the importance of environmental management in reducing vector density [16, 21]. Treatment adherence was suboptimal, with only 15.57% who completed full antimalarial doses and 39.07% who sought care at health facilities, mirroring findings in Ethiopia and Uganda regarding incomplete treatments and delayed care [20, 22].

Most participants (80.3%) reported the presence of a nearby health facility, yet 38% traveled 61–120 min to access care, highlighting geographic barriers to timely treatment [22]. Traditional healers remained a care option for 24.9%, reflecting cultural influences and cost considerations [20, 22]. Malaria diagnosis relied on microscopy (33.9%) and rapid diagnostic tests (28.7%), whereas drug availability (67.2%) and affordability (53.8%) were moderate, and preventive interventions such as ITN distribution (47.57%) and IRS (39.34%) were inconsistently implemented [16, 18, 20].

Self‐reported malaria prevalence was significantly higher in rural participants, consistent with studies showing rural communities face increased exposure due to proximity to breeding sites, inadequate housing, and limited vector control interventions [12, 23–25]. Consistent ITN use and elimination of breeding sites were associated with lower infection rates, reinforcing the effectiveness of these preventive strategies [26–28]. Spatial differences across woredas further underscore the need for geographically targeted interventions, particularly in hotspot areas such as Enarg Enawga, Jabi Tinan, and Dembecha [18, 29].

Proximity to health facilities, drug availability, and affordability did not significantly influence malaria prevalence, suggesting that behavioral and environmental factors may have stronger effects on transmission [27, 29].

## 5. Conclusion

Overall, the findings highlight that malaria remains a critical public health concern, particularly in rural communities, and underscore the importance of consistent vector control, environmental management, community education, and behavioral interventions to reduce disease burden.

## 6. Limitations of the Study

Because malaria history was self‐reported, the findings may be subject to recall bias; however, this was minimized by using a short recall period and structured questionnaires during data collection.

## Author Contributions

A.H. conceived the research title, collected the data, analyzed the data, and developed the draft of the manuscript, and L.F. revised the data analysis and the manuscript.

## Funding

No funding was received for this manuscript.

## Disclosure

All authors read and approved the final manuscript.

## Ethics Statement

Prior to data collection, ethical approval was obtained from the Ethical Review Committee of the Department of Biology at Debre Markos University.

## Consent

Informed consent was secured through an agreement between the researcher and the study participants. Consent for publication is not applicable.

## Conflicts of Interest

The authors declare no conflicts of interest.

## Data Availability

The data that support the findings of this study are available from the corresponding author upon reasonable request.
